# Enterolactone alters FAK-Src signaling and suppresses migration and invasion of lung cancer cell lines

**DOI:** 10.1186/s12906-016-1512-3

**Published:** 2017-01-09

**Authors:** Shireen Chikara, Kaitlin Lindsey, Pawel Borowicz, Melpo Christofidou-Solomidou, Katie M. Reindl

**Affiliations:** 1 Department of Biological Sciences, North Dakota State University, Fargo, ND 51808 USA; 2 Department of Medicine, University of Pennsylvania, Philadelphia, PA 19104 USA; 3 Department of Animal Sciences, North Dakota State University, Fargo, ND 51808 USA

**Keywords:** Enterolactone, Cell motility, F-actin, Flaxseed, Focal adhesion, Lung cancer cells, Rho GTPases

## Abstract

**Background:**

Systemic toxicity of chemotherapeutic agents and the challenges associated with targeting metastatic tumors are limiting factors for current lung cancer therapeutic approaches. To address these issues, plant-derived bioactive components have been investigated for their anti-cancer properties because many of these agents are non-toxic to healthy tissues. Enterolactone (EL) is a flaxseed-derived mammalian lignan that has demonstrated anti-migratory properties for various cancers, but EL has not been investigated in the context of lung cancer, and its anticancer mechanisms are ill-defined. We hypothesized that EL could inhibit lung cancer cell motility by affecting the FAK-Src signaling pathway.

**Methods:**

Non-toxic concentrations of EL were identified for A549 and H460 human lung cancer cells by conducting 3-(4, 5-Dimethylthiazol-2-yl)-2, 5-Dephenyltetrazolium Bromide (MTT) assays. The anti-migratory and anti-invasive potential of EL for lung cancer cell lines was determined by scratch wound healing and Matrigel® invasion assays. Changes in filamentous actin (F-actin) fiber density and length in EL-treated cells were determined using phalloidin-conjugated rhodamine dye and fluorescent microscopy. Vinculin expression in focal adhesions upon EL treatment was determined by immunocytochemistry. Gene and protein expression levels of FAK-Src signaling molecules in EL-treated lung cancer cells were determined using PCR arrays, qRT-PCR, and western blotting.

**Results:**

Non-toxic concentrations of EL inhibited lung cancer cell migration and invasion in a concentration- and time-dependent manner. EL treatment reduced the density and number of F-actin fibers in lung cancer cell lines, and reduced the number and size of focal adhesions. EL decreased phosphorylation of FAK and its downstream targets, Src, paxillin, and decreased mRNA expression of cell motility-related genes, RhoA, Rac1, and Cdc42 in lung cancer cells.

**Conclusions:**

Our data suggest that EL suppresses lung cancer cell motility and invasion by altering FAK activity and subsequent activation of downstream proteins needed for focal adhesion formation and cytoskeletal rearrangement. Therefore, administration of EL may serve as a safe and complementary approach for inhibiting lung tumor cell motility, invasion, and metastasis.

**Electronic supplementary material:**

The online version of this article (doi:10.1186/s12906-016-1512-3) contains supplementary material, which is available to authorized users.

## Background

Enterolactone (EL) is a mammalian lignan derived from the plant lignan secoisolariciresinol diglucoside (SDG) that shows anti-migratory effects for breast, colon, and prostate cancer cells with limited or no toxicity to healthy cells [1–6]. EL suppressed adhesion, motility, and invasion of breast cancer cells by remodeling the actin cytoskeleton, downregulated gene expression of matrix metalloproteinases (MMP-2, −9, and −14), and inhibited FAK signaling [4, 5]. However, it is not clear what effect EL has on lung cancer cell motility. Further, the anti-cancer mechanisms for EL have not been clearly established for lung cancer.

Support for the use of lignans such as SDG or EL in lung cancer therapy comes from research showing their protective effects for healthy lung tissues in lung injury models. SDG reduced murine lung inflammation and oxidative damage inflicted by radiation, a standard treatment for metastatic lung cancer [7–10]. Given the protective effects of lignans for healthy lung tissue, and their anti-migratory effects for other cancer types, we hypothesized that EL would inhibit lung cancer cell migration.

Focal adhesion kinase (FAK) and steroid receptor coactivator (c-Src) are signaling proteins that regulate cytoskeletal dynamics and cell motility by influencing actin polymerization and focal adhesion turn-over [11, 12]. FAK and Src expression are elevated in non-small cell lung cancer (NSCLC) tissues as compared to normal lung tissue, and positively correlate with advanced stages of disease [13–15]. Preclinical studies have shown that FAK and Src inhibitors effectively suppress lung cancer metastases; however, these agents induce cytotoxicity in healthy tissues [16, 17]. Therefore, there is a need for less toxic agents that target FAK-Src signaling and inhibit lung cancer cell motility.

The objective for this research was to identify EL as a less toxic agent to inhibit lung cancer cell motility, and to determine its anti-migratory mechanisms by focusing on FAK-Src signaling and down-stream effects. Our central hypothesis was that EL inhibits lung cancer cell motility by altering focal adhesion formation and F-actin structure by decreasing FAK-Src signaling. To address this hypothesis, we investigated the anti-migratory and anti-invasive effects of EL on NSCLC cells (A549 and H460), and determined the impact on focal adhesion formation, actin filaments, and the expression of mRNA and proteins associated with cell motility. Our findings suggest that EL or its parent lignan compound SDG could be used to inhibit NSCLC cell motility by influencing FAK-Src signaling.

## Methods

### Materials

Purified EL (99.2% pure) was purchased from ChromaDex, Inc (Santa Ana, CA). 3-(4, 5-Dimethylthiazol-2-yl)-2, 5-Dephenyltetrazolium Bromide (MTT) was obtained from AbD Serotech (Raleigh, NC). Matrigel® invasion chambers with 8 μm pore size inserts were obtained from BD Biosciences (Bedford, MA). Crystal violet was obtained from Alfa Aesar (Ward Hill, MA). Rhodamine phalloidin was purchased from Thermo Fisher Scientific (Waltham, MA). Antibodies for p-FAK^Tyr397^, t-FAK, p-paxillin^Tyr118^, t-paxillin, p-Src ^Tyr416^_,_ p-Src ^Tyr527^_,_ t-Src, RhoA, p-Rac/Cdc42, t-Rac/Cdc42, GAPDH, and anti-rabbit HRP-conjugated secondary antibody were purchased from Cell Signaling Technology (Danvers, MA). Antibody for vinculin was purchased from EMD Millipore (Billerica, MA). Ki-67 and anti-mouse Alexa 633 fluorophore-conjugated antibodies were purchased from Abcam (Cambridge, MA). DAPI was obtained from Biotium (Fremont, CA). Coverslips were obtained from Carl Zeiss (Ontario, Canada).

### Cell culture and drug treatment

The human NSCLC cell lines A549 and H460 were purchased from ATCC (Manassas, VA). Cells were maintained in RPMI-1640 medium (Sigma Aldrich; St. Louis, MO), with 10% (v/v) fetal bovine serum (FBS; Atlanta Biologicals; Flowery Branch, GA) and 1% penicillin/streptomycin (Thermo Fisher Scientific). The cell lines were incubated at 37 °C in a humidified atmosphere of 95% air and 5% carbon dioxide. They were sub-cultured by enzymatic digestion with 0.25% trypsin/1mM EDTA solution (Hyclone; Logan, UT) when they reached approximately 70% confluency.

EL was dissolved in 100% dimethysulfoxide (DMSO; Corning Cellgro®; Corning, NY) at a stock solution concentration of 200 mM. The stock solution was freshly diluted in PBS (pH 7.2) to desired concentrations for every experiment. The vehicle control was 0.2% DMSO.

### MTT assay

The effect of EL on the viability of A549 and H460 cells lines was studied using the MTT assay. Briefly, 3000 cells/well were plated in a 96-well plate. After 24 h of incubation, medium was removed and the cells were washed with PBS. Fresh RPMI-1640 medium was added, and the cells were treated with vehicle (DMSO) or various concentrations of EL (0, 10, 25, 50, 75, and 100 μM) for 24 and 48 h. Next, 10 μl MTT (0.5 mg/ml) was added to each well, and the plate was incubated for 4 h at 37 °C. Later, the medium was removed and the cells were washed with PBS. The resulting formazan crystals were dissolved in 100 μl of DMSO, and the absorbance reading of each well was taken at 570 nm using a plate reader (Microplate XMark™ spectrophotometer; Bio-Rad; Hercules, CA). The percentage of cell survival was calculated using the background-corrected absorbance as shown in the following formula:

$$ \mathrm{Cell}\ \mathrm{survival}\ \left(\%\right) = \left({\mathrm{Absorbance}}_{\mathrm{Treatment}}/{\mathrm{Absorbance}}_{\mathrm{Control}}\right)\ \mathrm{X}\ 100 $$

The data shown represent the mean and standard deviation from 8 replicate wells for each treatment for 3 independent experiments.

### Migration assay

The ability of EL to inhibit migration of A549 and H460 cells was investigated using a wound healing assay. Cells were seeded into 6-well dishes and grown to 80–90% confluency. A sterilized 10 μl pipette tip was used to generate a wound across the cell monolayer. The cellular debris was washed with PBS, serum-free RPMI-1640 medium was added to each well, and the cells were treated with fresh vehicle (DMSO) or EL (10, 50, and 100 μM) every 24 h. The open gap was photographed microscopically after 24 and 48 h. The migration ability of the cells was determined by measuring the width of the monolayer wound for three fields per treatments at 24 and 48 h after scraping, and the migration index was calculated using the following formula, where ‘T’ stands for time (24 or 48 h).

$$ \mathrm{Migration}\ \mathrm{index} = \left(\frac{\left."0"\ \mathrm{h}\ \mathrm{scratch}\ \mathrm{width}\ \hbox{--}\ "\mathrm{T}"\ \mathrm{h}\kern0.5em \mathrm{scratch}\ \mathrm{width}\right)}{0\ \mathrm{h}\ \mathrm{scratch}\ \mathrm{width}}\right)\ \mathrm{X}\kern0.5em 100 $$

The data shown represent the mean and standard deviation of 3 independent experiments.

### Ki-67 Immunocytochemistry

To verify that EL inhibited migration of lung cancer cells independent of its effects on cell proliferation, we stained lung cancer cells with the proliferation marker Ki-67. Cells were seeded into 6-well dishes, and after they reached 80–90% confluency, a wound across the cell monolayer was created with a sterilized 10 μl pipette. After removal of cellular debris, serum-free RPMI-1640 medium was added, and the cells were treated with vehicle (DMSO) or EL (100 μM) for 24 and 48 h, with fresh treatment added after 24 h. The cells were fixed with 4% formaldehyde solution and permeabilized with 0.1% Triton-X 100. Next, the cells were washed with 1x TBS-tween (0.1%) and blocked with 10% normal goat serum (NGS) for 1 h, washed with 1x TBS-tween (0.1%), and incubated with anti-Ki-67 primary antibody for 3 h. The cells were washed again with 1x TBS-tween (0.1%) and incubated with Alexa Fluor 633 anti-rabbit secondary antibody for 1 h. After DAPI counterstaining, the number of Ki-67 positive cells across the wound were examined using a Zeiss Axio Observer Z1 inverted microscope with LSM700 laser scanning unit and 20x 0.8 objective (Zeiss, Thornwood, NY). The cell proliferation ratio was calculated using ImagePro Premier software (Media Cybernetics, Silver Spring, MD, USA). The data shown represent the mean and standard deviation of 2 random fields per sample for three independent experiments for each cell line.

### Invasion assay

The ability of EL to inhibit invasion of A549 and H460 cells was investigated using Matrigel® invasion chambers consisting of invasion inserts (8 μm pore size). A549 and H460 cells (5 x 10^6^ cells/well), suspended in serum-free RPMI-1640, were placed in the upper chamber of the transwell inserts and incubated with vehicle (DMSO) or EL (100 μM). RPMI-1640 medium supplemented with 50% FBS was added to the lower chamber. The plates were incubated in a humidified atmosphere with 95 air and 5% CO_2_ at 37 °C for 24 and 48 h. Fresh serum-free medium along with EL treatment was added in the upper chamber after 24 h. The non-invasive cells, present on the inside of the upper chamber, were removed by wiping with a cotton swab dipped in PBS, and invasive cells, present on the underside of the upper chamber, were fixed with 4% formaldehyde in PBS and later stained with 2% crystal violet. Invasive cells were then photographed under a light microscope at 200X.

### Cytoskeleton organization analysis

Actin cytoskeleton staining was carried out with phalloidin conjugated-rhodamine dye. A549 and H460 cells, grown on glass coverslips, were treated with vehicle (DMSO) or EL (10, 50, and 100 μM) for 24 h. After treatment, the cells were washed twice with PBS and fixed with 4% formaldehyde in PBS for 15 min at room temperature. The cells were washed twice with PBS and permeabilized with 0.1% Triton-X 100 for 5 min. Following permeabilization, the cells were washed with PBS and blocked with 1% BSA for 1 h. The cells were then incubated with phalloidin conjugated-rhodamine for 20 min at 37 °C. After incubation, the cells were washed with PBS and incubated with DAPI for 3–4 min to label nuclei. Finally, coverslips were mounted onto slides with the help of aqua-poly-mount mounting medium. Confocal images were acquired using an inverted fluorescence microscope with 40X oil immersion lens. At least 5 independent random fields per sample were captured from three independent experiments. The acquired images were converted into binary images for quantification of the density and average length of F-actin fibers using Image Pro Premier software (9.0).

### Immunofluorescence and confocal microscopy

To visualize focal adhesions, A549 and H460 cells grown on fibronectin-coated coverslips were treated with vehicle (DMSO) or EL (100 μM) for 24 h. After treatment, the cells were washed with PBS, fixed with 4% formaldehyde solution for 15 min at room temperature, and permeabilized with 0.1% Triton-X 100 for 10 min. Next, the cells were washed with 1x TBS-tween (0.1%),blocked with 10% NGS for 1 h, washed with 1x TBS-tween (0.1%), and incubated with anti-vinculin primary antibody for 3 h. The cells were washed again with 1x TBS-tween (0.1%) and incubated with Alexa Fluor 633 anti-mouse secondary antibody for 1 h. These cells were then incubated with DAPI for 3–4 min to label nuclei. The coverslips were then mounted onto slides with the help of aqua-poly-mount mounting medium and examined using Zeiss Axio Observer Z1 inverted microscope with LSM700 laser scanning unit and 40x 1.3NA oil objective (Zeiss, Thornwood, NY). The number and size of focal adhesions per cell were examined analyzed using the ImagePro Premier software (Media Cybernetics, Silver Spring, MD, USA) in 10 individual cells for each treatment for three independent experiments.

### Microarray analysis for cell-motility related genes

Total RNA was isolated from untreated control and EL-treated (100 μM; 24 h) A549 cells (1 X 10^6^ cells) using the Fisher SurePrep kit as per the manufacturer’s instructions. cDNA was synthesized using 100 ng of total RNA and the qScript cDNA synthesis kit (Quanta Biosciences; Beverly, MD). Subsequently, qPCR was performed using PerfeCTa SYBR Green FastMix (Quanta Biosciences) and the PrimePCR^TM^ Pathway Plates (Bio-Rad; Hercules, CA) to investigate the effects of EL on cell motility-related genes regulating FAK and platelet-derived growth factor (PDGF) signaling. The cycling parameters were: 95 °C for 10 min followed by 40 cycles at 95 °C for 30 s and 60 °C for 1 min, and a dissociation program that included 95 °C for 1 min, 55 °C for 30 s, and 95 °C for 30 s ramping up at 0.2 °C/s. One distinct peak was observed for each primer set, suggesting target specificity. Duplicate wells were run for each experiment, and the experiment was performed in duplicate. The relative change in gene expression was calculated using 2^-ΔΔCt^ method using housekeeping genes TBP, GAPDH, and HPRT1 as internal controls.

### Quantitative reverse transcriptase polymerase chain reaction (qRT-PCR)

Total RNA was isolated from untreated control and EL-treated (100 μM; 24 h) A549 and H460 cells (1 X 10^6^ cells) using the Fisher SurePrep Kit (Waltham, MA) as per the manufacturer’s instructions. cDNA was synthesized using 100 ng of total RNA and the qScript cDNA synthesis kit (Quanta Biosciences). Primers for RhoA, Rac, Cdc42, and 18S rRNA were designed using Primer Express software (version 2.0, Applied Biosystems; Foster City, CA), and were synthesized by Integrated DNA Technologies (Coralville, IA). The primer sequences were as follows: RhoA Forward GAGTTGGCTTTGTGGGACACA, RhoA Reverse ACTATCAGGGCTGTCGATGGA, Rac1 Forward GCTTATGGGATACAGCTGGACAA, Rac1 Reverse AGGACTCACAAGGGAAAAGCAA, Cdc42 Forward GATTACGACCGCTGAGTTATCCA, Cdc42 Reverse CAGGCACCCACTTTTCTTTCAC.

Steady-state mRNA levels for the cell cycle-related genes were evaluated by qPCR using PerfeCTa SYBR Green FastMix (Quanta Biosciences). The cycling parameters were: 95 °C for 10 min followed by 40 cycles at 95 °C for 30 s and 60 °C for 1 min, and a dissociation program that included 95 °C for 1 min, 55 °C for 30 s, and 95 °C for 30 s ramping up at 0.2 °C/s. One distinct peak was observed for each primer set, suggesting target specificity. Duplicate wells were run for each experiment and the experiment was performed in triplicate. The relative change in gene expression was calculated using 2^-ΔΔCt^ method using the housekeeping gene 18S rRNA as an internal control.

### Western blotting

A549 and H460 lung cancer cells (3 x 10^5^ cells/well) were seeded in a 24-well plate. After 24 h of incubation, the cells were treated with vehicle (DMSO) or EL (100 μM) for 0, 1, 3, 6, 12, and 24 h. The cells were then harvested by trypsinization, centrifuged at 300 x g for 10 min. The resulting cell pellet was then lysed by brief sonication in 100 μl of SDS lysis buffer (Cell Signaling Technologies) containing protease and phosphatase inhibitors (Roche; Indianapolis, IN) to dissociate cell membranes. Fifty micrograms of total protein isolated from these cells were electrophoresed on 7.5% SDS-polyacrylamide gels at 100 V for 1 h. Proteins were then transferred to nitrocellulose membranes at 100 V at 4 °C for 70 min. The blots were then probed overnight at 4 °C with primary antibodies (1:1000) for p-FAK^Tyr397^, t-FAK, p-paxillin^Tyr118^, t-paxillin, p-Src^Tyr416^_,_ p-Src^Tyr527^_,_ t-Src, RhoA, p-Rac/Cdc42, t-Rac/Cdc42, and GAPDH. The next day, the blots were rinsed with 1X TBS-tween (0.1%) and probed with anti-rabbit HRP-conjugated secondary antibodies (1:5000) for 1 h at room temperature. The western blots were analyzed using SuperSignal West Pico Chemiluminescent Substrate (Thermo Fisher Scientific) and the images were captured using the MultiImage™ Light Cabinet (Alpha Innotech; San Leandro, CA). Western blotting was performed in triplicate. The densitometry results were obtained using ImageJ software.

### Statistical analyses

Data are presented as means ± standard deviation for at least 3 independent experiments. The statistical significance of difference between the control and treatment groups was determined by paired *t*-test or two-way ANOVA. *p ≤ 0.05* were considered statistically significant.

## Results

### EL has minimal effect on growth of lung cancer cells at 24 and 48 h

**Fig. 1:**
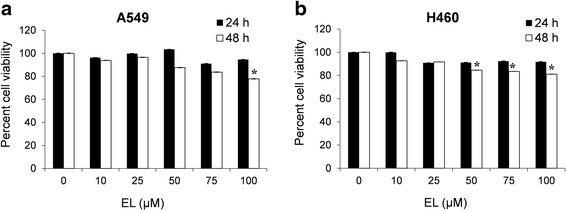
EL has minimal effects on lung cancer cell viability. Lung cancer cell lines **a** A549 and **b** H460 were treated with different concentrations of EL (0, 10, 25, 50, 75, and 100 μM) for 24 and 48 h, and cell viability was measured using an MTT assay. The data represent the average ± standard deviation of eight replicate wells for three independent experiment for each cell line. *p ≤ 0.05* was considered statistically significant when compared with untreated control

To investigate the effect the EL on lung cancer cell viability, A549 and H460 cells were treated with different concentrations of EL (0–100 μM) for 24 and 48 h. Viability was assessed using the MTT assay. The results show that EL had no effect on the proliferation of A549 and H460 cells at 24 h (Fig. 1a and b). A minimal concentration-dependent decrease in cell proliferation was observed in response to EL-treatment at 48 h, with a 20% decrease observed in A549 and 15% decrease in H460 treated with the highest concentration (100 μM) of EL (Fig. 1a and b).

### EL inhibits in vitro migration of lung cancer cells

**Fig. 2:**
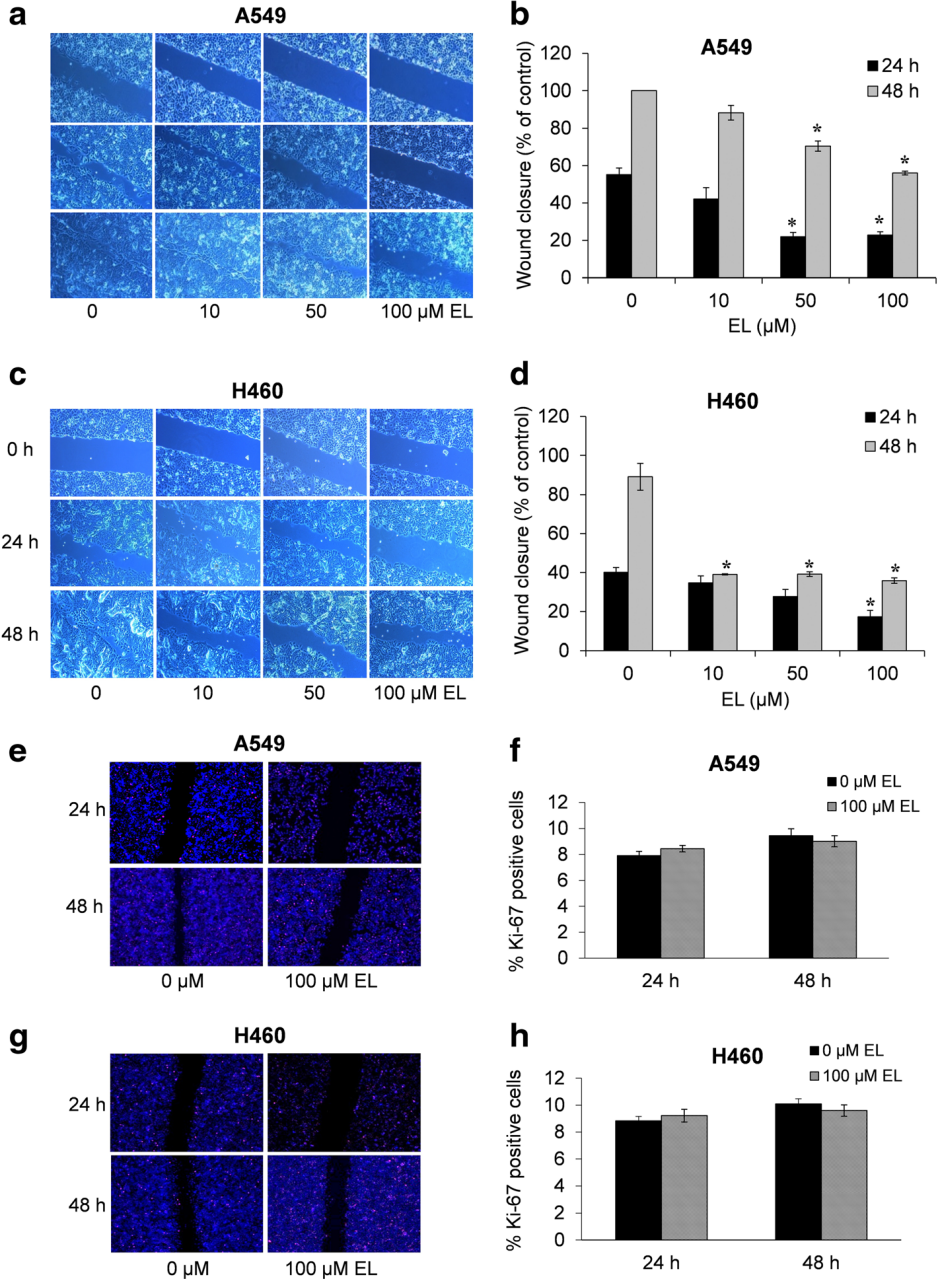
EL impairs the in vitro migration potential of lung cancer cells independent of cell proliferation. **a** A549 and **c** H460 cells were grown to 90% confluency in cell culture dishes. A scratch/wound was made in each dish. The cells were then treated with 0, 10, 50, and 100 μM EL for 24 or 48 h. Images were taken at each time point for the respective control and treatment groups. The distance across the wound was measured for three replicate experiments for **b** A549 and **d** H460 cells and quantified as the % migration index. Ki-67 staining and quantification of **e** and **f** A549 and **g** and **h** H460 cells were performed to identify the % of Ki-67 positive cells near the wound. The data represent the average ± standard deviation % migration index for three fields per treatments for three independent experiments. *p ≤ 0.05* was considered statistically significant when compared with untreated control

A scratch wound healing assay was used to examine the anti-migratory effects of EL in A549 and H460 cells. Cells were either treated with vehicle control (DMSO) or EL (10, 50, 100 μM) for 24 and 48 h. Control A549 and H460 cells demonstrated their migration potential by resulting in 55 and 40% wound repair after 24 h, and 100 and 90% wound repair after 48 h, respectively (Fig. 2). On the other hand, EL treatment (10, 50, and 100 μM) of A549 cells suppressed wound healing in a concentration- and time- dependent manner, with 42, 22, and 23% wound closure after 24 h, respectively (Fig. 2a and b), and 88, 70, and 56% wound closure after 48 h, respectively (Fig. 2a and b). For H460 cells, 10, 50, and 100 μM EL treatment resulted in 35, 28, and 17% wound closure after 24 h (Fig. 2c and d), respectively, and 39, 39, and 36% of wound closure after 48 h (Fig. 2c and d), respectively.

Ki-67 immunocytochemistry was performed to rule out the possibility that EL inhibited cell proliferation which resulted in reduced cell migration. After creating a scratch wound, A549 and H460 cells were either treated with the vehicle control (DMSO) or EL (100 μM) for 24 and 48 h. The cell proliferation rate, qualitatively measured by the number of Ki-67 positive cells near the wound edge, was similar in control and EL-treated A549 and H460 cells (Fig. 2e and g). In A549 cells, on an average, there were 7.9% Ki-67 positive cells in the control group and 8.4% in the treatment group after 24 h, and 9.4 and 9.0%, respectively, after 48 h (Fig. 2f). Similarly, in H460, these numbers were 8.8 and 9.2% after 24 h, and 10.1 and 9.5% after 48 h (Fig. 2h). These results suggest that the anti-migratory effects of EL on lung cancer cells are independent of its effect on cell proliferation.

### EL inhibits in vitro invasion of lung cancer cells

**Fig. 3:**
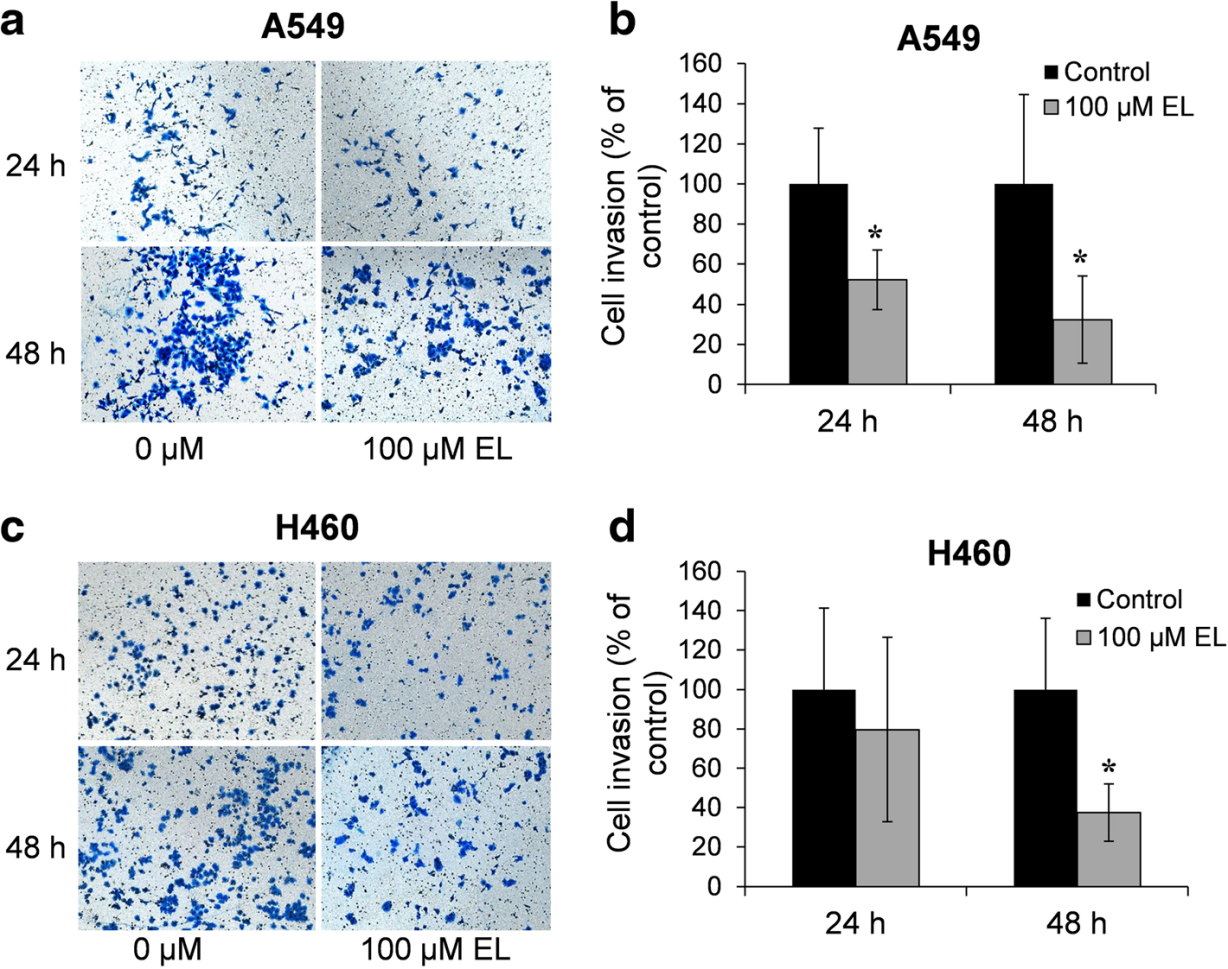
EL suppresses in vitro invasive potential of lung cancer cells. **a** A549 and **c** H460 cells were placed in the upper chamber of the Matrigel inserts (8 μm pore size) in serum-free RPMI-1640 medium, and then treated with 0 or 100 μM EL for 24 or 48 h. The number of invaded cells were fixed with 4% formaldehyde and stained with 2% crystal violet. Invasive cells were then photographed under a light microscope at 200x. The number of invasive **b** A549 and **d** H460 cells were counted for three replicate experiments and quantified. *p ≤ 0.05* was considered statistically significant when compared with untreated control

The effect of EL treatment (100 μM) on cancer cell invasion after 24 and 48 h was studied using Matrigel® invasion chambers. As shown in Fig. 3a and c, compared to the untreated control, the number of invading cells represented by crystal-violet stain was reduced remarkably in a time-dependent manner in A549 and H460 cells. The inhibitory effects of EL (100 μM) on cancer cell invasion, as measured by the percentage of control, were 47 and 68% in A549 cells, and 20 and 37% in H460 cells, after 24 and 48 h, respectively (Fig. 3b and d). These results suggest that EL inhibits the in vitro invasive potential of lung cancer cells.

### EL affects the actin cytoskeleton in lung cancer cells

**Fig. 4:**
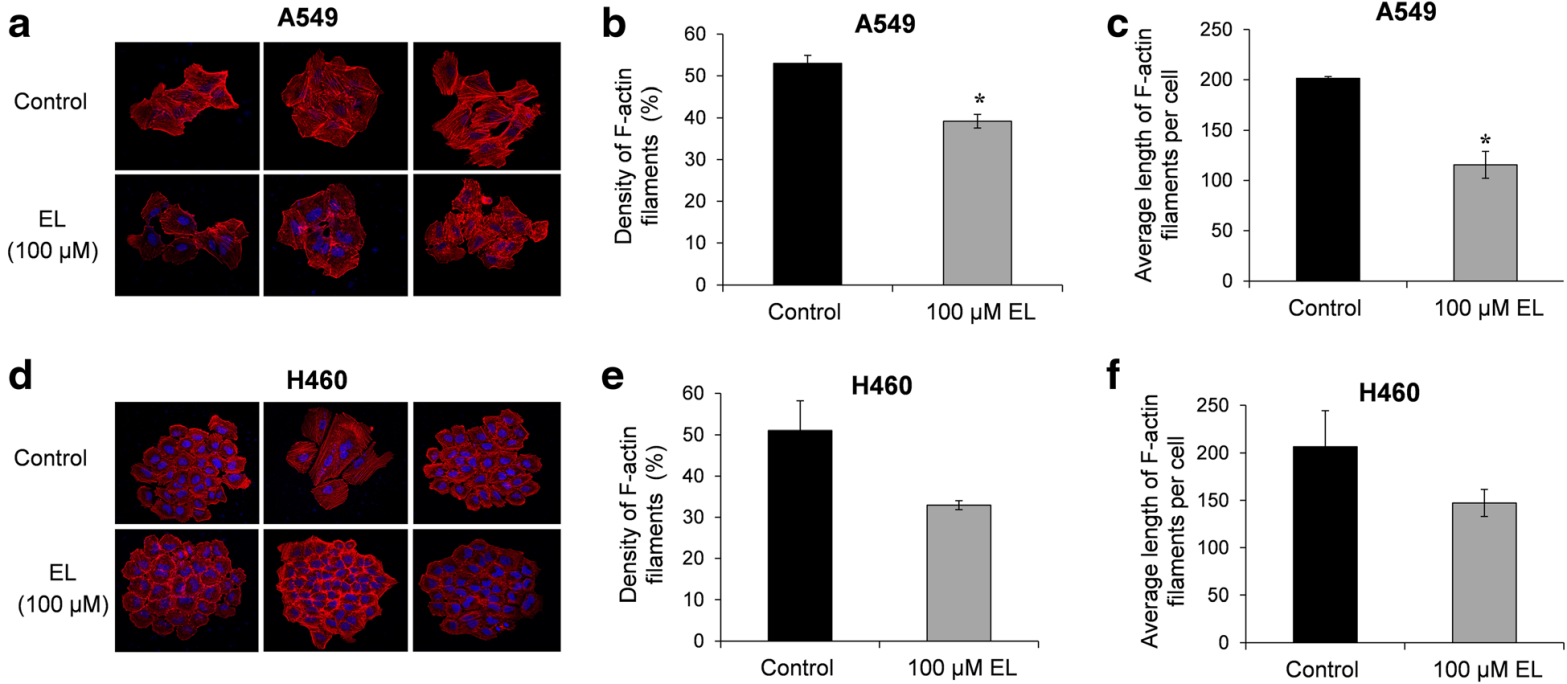
EL effects cytoskeletal organization. **a** A549 and **d** H460 lung cancer cells grown on glass coverslips were treated with DMSO or EL (100 μM) for 24 h. Immunocytochemistry was conducted using rhodamine-conjugated phalloidin to visualize F-actin fibers. Cells were imaged using an inverted fluorescence microscope (blue fluorescence represents DAPI stained nuclei and red fluorescence indicates rhodamine-conjugated phalloidin-stained F-actin filaments). The acquired images were converted into binary images for quantification on Image Pro Premier software (9.0). The density of F-actin filaments was determined for both **b** A549 and **e** H460 cells treated with or without EL. The average length of the F-actin filaments per cell was determined for **c** A549 and **f** H460 cells. *p ≤ 0.05* was considered statistically significant when compared with untreated control

Given the importance of the cytoskeletal structure on cell motility, the effect of EL treatment (100 μM; 24 h) on the distribution of F-actin fibers was visualized with the help of phalloidin-conjugated rhodamine dye. The results from immunofluorescence microscopy indicate that after 24 h, control A549 and H460 cells exhibited dense F-actin fibers, while EL-treated cells showed a loss of F-actin fibers (Fig. 4a and d). Further, long F-actin fibers running across the cell body were seen in control cells, while branched and broken actin fibers were observed in EL-treated cells (Fig. 4a and d). Compared to the control, EL treatment resulted in a decrease in the percentage of polymerized F-actin fibers from 52.9 to 39.2% in A549 cells and from 50.9 to 32.9% in H460 cells (Fig. 4b and e). This difference was statistically significant in A549 cells (*p ≤ 0.05)*. In addition, compared to the control, EL-treatment decreased the average length of F-actin fibers from 201.4 to 115.5 nm in A549 and 206.5 to 147.3 in H460 cells, with a statistically significant difference observed in A549 cells (*p ≤ 0.05)* (Fig. 4c and f). These results suggest that EL inhibits lung cancer cell motility by interfering with actin microfilament formation.

### EL reduces the number and size of focal adhesions in lung cancer cells

**Fig. 5:**
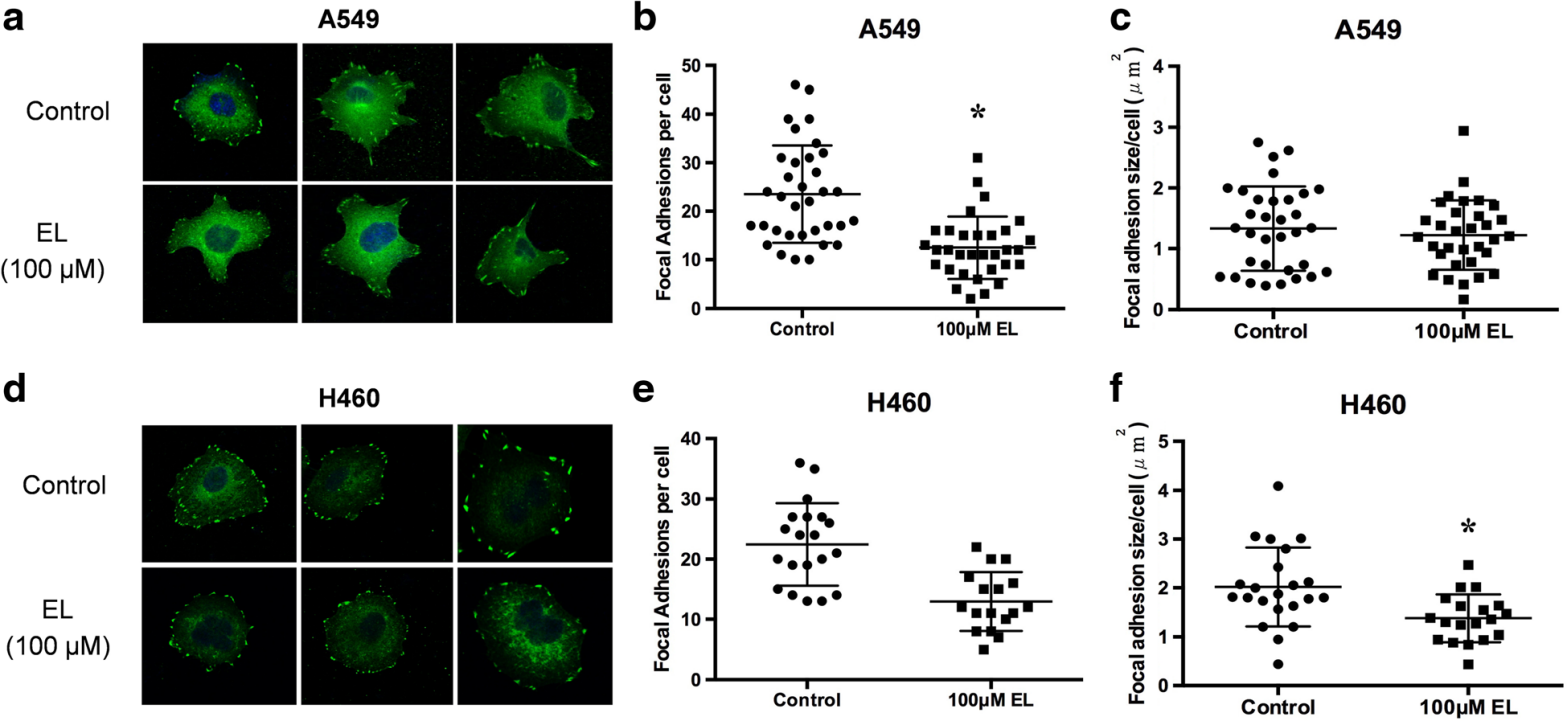
EL reduces the number and size of focal adhesions in lung cancer cells. A549 and H460 lung cancer cells grown on glass coverslips were treated with DMSO or EL (100 μM) for 24 h. Immunocytochemistry for vinculin expression and localization was performed for **a** A549 and **d** H460 cells. Cells were imaged using an inverted fluorescence microscope. Image Pro Premier software (9.0) was used to identify the number of focal adhesions per cell for **b** A549 and **e** H460 cells. The focal adhesion size (μm^2^) per cell was also determined for **c** A549 and **f** H460 cells based on pixel count

Focal adhesions provide a structural link between the actin cytoskeleton and extracellular matrix (ECM). Therefore, we investigated the effect of EL on focal adhesion number and size in lung cancer cells. A549 and H460 cells were seeded onto fibronectin-coated coverslips, and immunofluorescent staining for expression of vinculin, a membrane-cytoskeletal protein present in focal adhesion plaques, was performed. Control and EL-treated (100 μM; 24 h) A549 and H460 cells displayed prominent focal adhesions at their periphery (Fig. 5a and d). However, EL-treatment caused a reduction in the average number of focal adhesions per cell for both A549 and H460 cells. In A549 cells, control cells exhibited an average of 21.6 focal adhesions, while EL-treated cells showed a statistically significant reduction in focal adhesions with an average of only 12.5 focal adhesions per cell (*p ≤ 0.05)* (Fig. 5b). Similarly, in H460 cells, the control cells exhibited an average of 22.5 focal adhesions, while EL-treated cells showed a reduction in focal adhesion with an average of 13.1 focal adhesions per cell (Fig. 5e). In addition, EL treatment affected the size of focal adhesions in A549 and H460 cells. In A549 cells, the average size of focal adhesions was 1.33 μm^2^/cell in control cells and 1.22 μm^2^/cell in EL-treated cells (Fig. 5c). Similarly, in H460 cells, the average size of focal adhesions was 2.02 μm^2^/cell while EL-treated cells showed a statistically significant decrease in the size of focal adhesions with the average being 1.38 μm^2^/cell (*p ≤ 0.05)* (Fig. 5f).

### EL influences the mRNA expression of cell motility-related genes

**Fig. 6:**
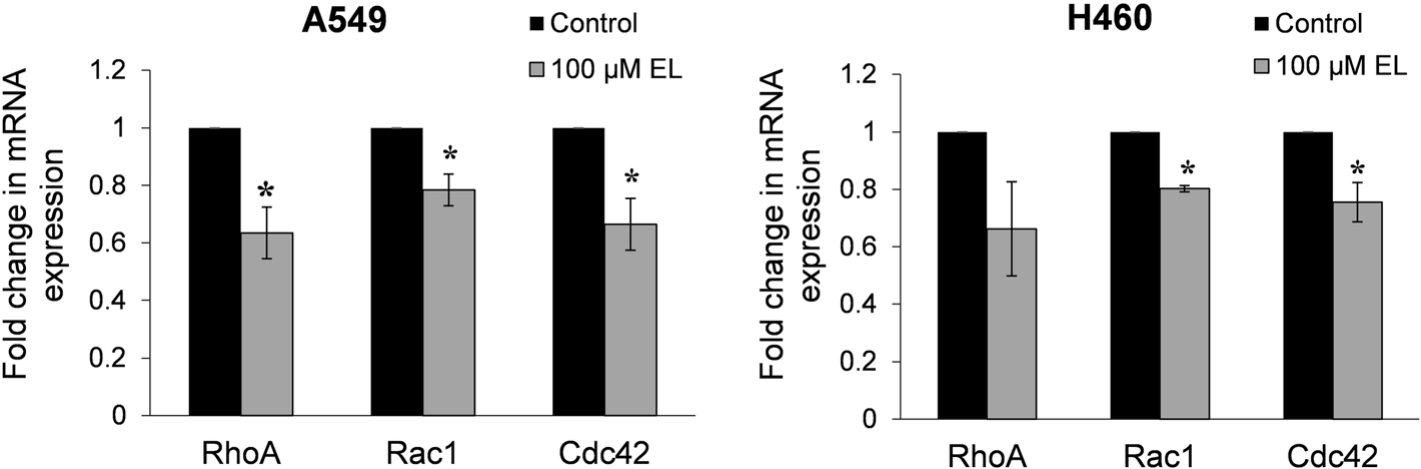
EL treatment of lung cancer cells results in decreased mRNA expression of Rho GTPases. **a** A549 and **b** H460 cells were treated with 0 or 100 μM EL for 24 h. Total RNA was extracted from the cells and subjected to qRT-PCR for RhoA, Rac1, and Cdc42. The experiments were conducted in triplicate. The data were normalized to 18S rRNA expression and represent the average fold change in mRNA expression for EL-treated cells relative to the control. *p ≤ 0.05* was considered statistically significant when compared with untreated control

We employed PrimePCR™ Pathway microarray plates to investigate the effects of EL on the expression of genes associated with FAK and PDGF signaling pathways. We found that EL-treatment (100 μM; 24 h) led to differential regulation of a number of genes in the two signaling pathways (Additional file 1: Table S1). Importantly, we observed that RhoA, Rac1, and Cdc42 genes that overlapped in the two signaling pathways, were consistently down-regulated in response to EL-treatment. Further, an integrin subunit alpha 2 (ITGA2) that helps anchor cells to the ECM was significantly up-regulated. To validate the results obtained by microarray analysis, we performed RT-qPCR. Compared to the control, EL-treatment (100 μM; 24 h) led to down-regulation of RhoA, Rac1, and Cdc42 mRNA expression in A549 and H460 cells (Fig. 6a and b). The observed decrease in mRNA expression for all the three genes was statistically significant in A549 cells, while H460 cells showed a significant (*p ≤ 0.05*) decrease in Rac1 and Cdc42 mRNA expression. These results suggest that EL treatment alters the expression of key transcripts associated with cell motility.

### EL modulates FAK-Src signaling in lung cancer cells

**Fig. 7:**
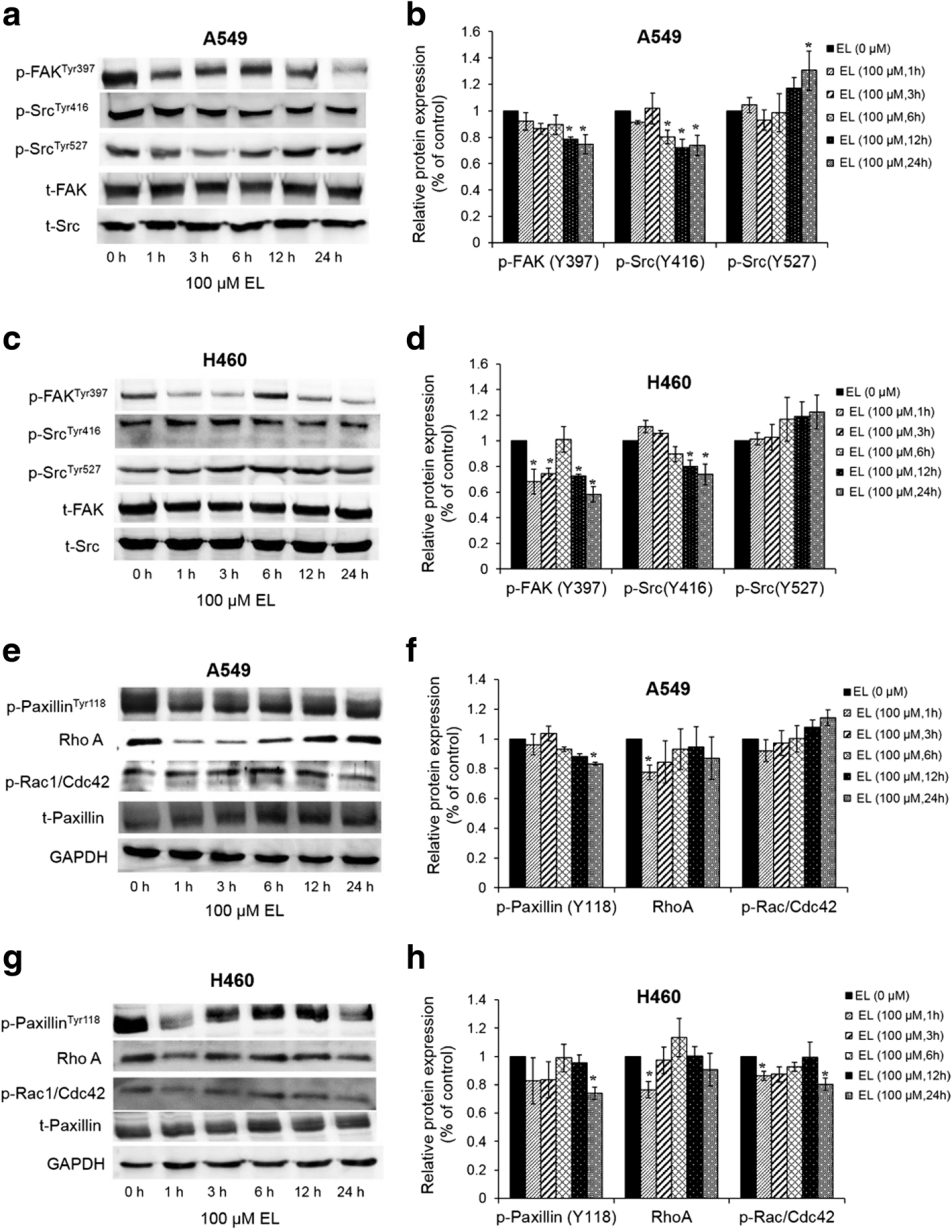
EL treatment of lung cancer cells alters levels of FAK-Src signaling proteins. **a**-**d** A549 or **e**-**h** H460 cells were treated with 100 μM EL for 0, 1, 3, 6, 12, or 24 h. Western blotting was done to identify changes in total or phosphorylated levels of FAK, Src, paxillin, RhoA, and Rac/Cdc42 proteins. The blots represent one typical result from three independent experiments. Phosphorylated levels of proteins were normalized to their respective total protein levels. Alternatively, GAPDH was used as a loading control. Densitometry was performed using ImageJ software. *p ≤ 0.05* was considered statistically significant when compared with untreated control

De-regulation of the FAK-Src signaling cascade mediates cancer cell migration in lung cancer cells. Therefore, we investigated the effect of EL treatment on the phosphorylation status of FAK and Src proteins. The western blot results show that 100 μM EL decreased phosphorylation of FAK on Y397 (Fig. 7a-d). In addition, in A549 and H460 cell, 100 μM EL decreased phosphorylation of Src on its kinase domain containing an auto-phosphorylation site (Y416) that is necessary for its activation (Fig. 7a-d). Simultaneously, EL increased phosphorylation on the carboxyl terminal domain of Src that contains a regulatory tyrosine (Y527), that upon phosphorylation maintains Src in an inactive conformation (Fig. 7a-d). These results suggest that EL inhibits FAK-Src activation which could explain the observed changes in cell migration and invasion in A549 and H460 lung cancer cells.

### EL inhibits phosphorylation of paxillin and Rho proteins in lung cancer cells

Paxillin is a major substrate of the FAK-Src complex and plays an important role in cell migration and cytoskeletal reorganization. Phosphorylated FAK initiates phosphorylation of paxillin on Y118, a prerequisite for cell migration. Therefore, we investigated if inhibition of FAK-Src signaling pathway by EL as seen in Fig. 7a-d was accompanied by inhibition in paxillin phosphorylation. EL-treatment (100 μM) decreased phosphorylation status of paxillin on the Y118 residue in A549 and H460 cells (Fig. 7e-h).

RhoA, Rac1, and Cdc42 are critical proteins downstream of FAK-Src-paxillin signaling pathway and play important roles in cell migration. Therefore, we next investigated if EL-mediated downregulation in mRNA expression of RhoA, Rac1, and Cdc42 co-related with a decrease in their protein expression. As shown in Fig. 7e-h, decreased RhoA protein levels were observed in A549 and H460 cells after EL treatment (100 μM). EL treatment (100 μM) resulted in a slight increase in proteins levels of p-Rac/Cdc42 in A549 cells, and a decrease in p-Rac/Cdc42 protein levels in H460 (Fig. 7e-h). These results suggest that EL may affect stress fiber formation (a role of RhoA), but to a lesser degree filapodia or lamellopodia formation (roles for Rac and Cdc42).

## Discussion

Our data support the hypothesis that EL modulates lung cancer cell motility by inhibiting FAK-Src signaling. In this study, we observed that the anti-migration and invasion effects of EL in lung cancer cells were not dependent on its anti-proliferative effect. Here we have shown evidence that EL, at non-toxic concentrations, inhibits A549 and H460 cell invasion and migration by: (i) disrupting F-actin cytoskeleton dynamics, (ii) reducing the number and size of focal adhesions, and (iii) inhibiting the activation of the FAK-Src-paxillin signaling cascade and expression of down-stream motility regulators. These results are significant because inhibition of lung cancer metastasis remains a therapeutic challenge. Natural products, such as EL, that show anti-migratory effects with limited or no toxicity to healthy tissues would be valuable additions to lung cancer treatment regimens.

A number of studies have demonstrated the anti-proliferative effects of EL in cancer cell lines. In PC-3 prostate cancer cells, EL (40 μM; 24 h) inhibited IGF-1-induced cell proliferation by arresting cells at G0/G1-phase of the cell cycle and EL (60 μM; 20 h) suppressed migration [2]. Similarly, in MDA-MB-231 breast cancer cells, EL (100 μM) suppressed migration at 24 h and arrested cells at S-phase of the cell cycle after 48 h [5]. We have previously observed that EL (≥50 μM) inhibits proliferation of lung cancer cells and induces G0/G1- phase cell cycle arrest in lung cancer cells after 48 h (under review). In this study, we observed that non-toxic concentrations of EL (10, 50, and 100 μM) inhibited motility of lung cancer cells after only 24 h. Further, Ki-67 staining results indicate that EL-mediated inhibition of migration was not influenced by inhibition of cell proliferation. Therefore, our data supports previous research highlighting the cytostatic and anti-migratory potentials of EL, and extends the research to lung cancer, which has a high metastatic potential.

The majority of studies involving the anti-migration and invasion potential of EL have been performed using breast cancer cell lines, namely MDA-MB-231 cells [1, 4, 5]. Studies have shown that EL inhibits breast cancer cell adhesion to ECM proteins [1], and inhibits breast cancer cell migration and invasion by reducing MMP-2, −9, and −14 mRNA expression [4]. We did not detect significant changes in MMP mRNA or protein expression in A549 or H460 lung cancer cells treated with EL (data not shown). A likely explanation for differing molecular responses may stem from the fact that EL is derived from a phytoestrogen known as SDG, and may show varied effects on different cancer cell types.

A recent study provided more mechanistic detail for EL’s anti-migratory effects for breast cancer. This study demonstrated that EL inhibited breast cancer cell migration and invasion by decreasing levels of phosphorylated FAK at tyrosine 397 (Y397) and phosphorylated paxillin [5]. Phosphorylation of FAK at Y397 is critical for its activation and recruitment of Src [18]. Similarly, we found that EL reduced FAK Y397 phosphorylation and decreased phosphorylated paxillin in lung cancer cell lines. Further, we found EL elevated phospho-Src at Y527, and reduced phospho-Src at Y416. Phosphorylation at Y527 maintains Src in an inactive conformation and inhibits its recruitment to FAK, while phosphorylation at Y416 results in its activation [19, 20]. Deregulation of FAK-Src signaling is seen in several tumor types [21–23]. Lung tumors with elevated FAK and Src activity have increased metastatic potential [14, 15]. Therefore, inhibition of FAK-Src signaling by EL may reduce the capacity of lung cancer cells to migrate.

Cell migration is primarily driven by: (i) polymerization of F-actin, and formation of focal adhesion complexes at the leading edge, and (ii) F-actin depolymerization, and disassembly of focal adhesion complexes at the rear end of the cell. The activated FAK-Src-paxillin complex contributes to cell migration through the Rho family of small GTPases, such as RhoA, Rac1, and Cdc42 [24]. RhoA affects cell-cell or cell-ECM interaction by inducing cytoskeleton changes; Rac1 plays a role in membrane ruffling by driving actin polymerization; and Cdc42 is involved in the formation of filopodia through initiating F-actin filament assembly. In this study, we have shown that EL decreased the density of F-actin fiber and disrupted the formation of long stress fiber that traverse the cell body. This change in actin cytoskeleton leading to reduced cell migration was associated with down-regulation of RhoA, Rac1, and Cdc42 expression, which might impair cell body contraction and retraction at the rear end of the cell, and limit formation of protrusions at the leading edge.

In addition to down-regulating Rho GTPases, we found EL elevated ITGA2 mRNA expression in lung cancer cells. Integrins are transmembrane proteins that connect the extracellular matrix with the actin cytoskeleton. Loss of ITGA2 expression is frequently observed in solid tumors, and the silencing of ITGA2 resulted in enhanced breast cancer migration [25]. Therefore, EL-induced ITGA2 expression may anchor lung cancer cells to the matrix and prevent cell migration.

## Conclusions

In summary, EL treatment inhibits lung cancer cell migration by altering F-actin dynamics, suppressing formation of focal adhesion complexes, and activating FAK-Src signaling. EL also alters the expression of a number of regulators of the actin cytoskeleton and cell migration. Additional studies are needed to identify how EL could be used as a complementary approach to currently used chemotherapies for lung cancer. Also, more studies designed to investigate the mechanisms behind EL’s anti-cancer effects are needed. Altogether, EL holds promise as an adjuvant treatment to prevent tumor cell motility.

## Additional file

Additional file 1: Table S1. Microarray analysis for cell-motility related genes in FAK and PDGF signaling pathways. The fold change ± standard deviation in mRNA expression for the target genes in EL-treated A549 cells is shown relative to control-treated cells. (DOCX 14 kb)

## Abbreviations

c-Src: Steroid receptor coactivator
DMSO: Dimethylsulfoxide
ECM: Extracellular matrix
EL: Enterolactone
F-actin: Filamentous actin
FAK: Focal adhesion kinase
FBS: Fetal bovine serum
ITGA2: Integrin subunit alpha 2
MMPs: Matrix metalloproteinases
MTT: 3-(4, 5-Dimethylthiazol-2-yl)-2, 5-Dephenyltetrazolium Bromide
NGS: Normal goat serum
NSCLC: Non-small cell lung cancer
PDGF: Platelet-derived growth factor
qRT-PCR: Quantitative reverse transcriptase polymerase chain reaction
SDG: Secoisolariciresinol diglucoside
