# Supplementary material for: Enterolactone alters FAK-Src signaling and suppresses migration and invasion of lung cancer cell lines
Source: BMC Complement Altern Med. 2017 Jan 9;17:30. doi: 10.1186/s12906-016-1512-3 (PMC5223372; doi:10.1186/s12906-016-1512-3)
Supplement: Additional file 1: Table S1. — Microarray analysis for cell-motility related genes in FAK and PDGF signaling pathways. The fold change ± standard deviation in mRNA expression for the target genes in EL-treated A549 cells is shown relative to control-treated cells. (DOCX 14 kb) [file 12906_2016_1512_MOESM1_ESM.docx]

Additional file 1: Table S1. Microarray analysis for cell-motility related genes in FAK and PDGF signaling pathways. The fold change ± standard deviation in mRNA expression for the target genes in EL-treated A549 cells is shown relative to control-treated cells.

|  | FAK signaling | |  | | |  | PDGF signaling | |  | |
| --- | --- | --- | --- | --- | --- | --- | --- | --- | --- | --- |
| Gene Symbol | | Fold Change | |  | Gene Symbol | | | Fold Change | |  |
| AKT1 | | 0.770 ± 0.17 | |  | CDC42 | | | 0.706 ± 0.01 | |  |
| AKT2 | | 0.996 ± 0.06 | |  | PIK3CA | | | 1.370 ± 0.18 | |  |
| AKT3 | | 1.828 ± 0.33 | |  | PAK1 | | | 1.318 ± 0.08 | |  |
| CDC42 | | 0.532 ± 0.00 | |  | PIK3CB | | | 1.130 ± 0.04 | |  |
| CALM1 | | 0.841 ± 0.15 | |  | PDGFA | | | 1.090 ± 0.00 | |  |
| CALM2 | | 1.034 ± 0.22 | |  | PIK3CD | | | 1.112 ± 0.16 | |  |
| CALM3 | | 1.046 ± 0.20 | |  | PDGFB | | | 0.617 ± 0.08 | |  |
| CAMK2G | | 1.109 ± 0.16 | |  | PIK3R1 | | | 1.008 ± 0.05 | |  |
| CCND3 | | 0.433 ± 0.15 | |  | PDGFC | | | 1.119 ± 0.01 | |  |
| FN1 | | 2.028 ± 0.15 | |  | PDGFD | | | 0.830 ± 0.04 | |  |
| GRB2 | | 1.248 ± 0.16 | |  | PDGFRA | | | 1.152 ± 0.16 | |  |
| GSK3B | | 1.105 ± 0.17 | |  | PDGFRB | | | 1.241 ± 0.23 | |  |
| HRAS | | 0.810 ± 0.13 | |  | RAC1 | | | 0.818 ± 0.02 | |  |
| ITGA2 | | 2.396 ± 0.11 | |  | RhoA | | | 0.757 ± 0.02 | |  |
| ITGB1 | | 1.567 ± 0.17 | |  | TIAM1 | | | 0.826 ± 0.20 | |  |
| ITPR1 | | 2.530 ± 0.10 | |  | VAV1 | | | 0.746 ± 0.02 | |  |
| MAP2K1 | | 1.093 ± 0.16 | |  |  | | |  | |  |
| MAP2K2 | | 0.921 ± 0.36 | |  |  | | |  | |  |
| MAPK1 | | 0.909 ± 0.19 | |  |  | | |  | |  |
| MAPK3 | | 1.011 ± 0.05 | |  |  | | |  | |  |
| MAPK8 | | 1.304 ± 0.07 | |  |  | | |  | |  |
| PIK3CB | | 1.120 ± 0.07 | |  |  | | |  | |  |
| PIK3CD | | 1.126 ± 0.03 | |  |  | | |  | |  |
| PIK3R1 | | 1.140 ± 0.18 | |  |  | | |  | |  |
| PLCG1 | | 0.932 ± 0.08 | |  |  | | |  | |  |
| PTEN | | 1.309 ± 0.12 | |  |  | | |  | |  |
| PTK2 | | 1.095 ± 0.12 | |  |  | | |  | |  |
| PAK1 | | 1.322 ± 0.02 | |  |  | | |  | |  |
| PXN | | 0.844 ± 0.25 | |  |  | | |  | |  |
| PIK3CA | | 1.465 ± 0.12 | |  |  | | |  | |  |
| RAF1 | | 0.979 ± 0.06 | |  |  | | |  | |  |
| RAP1A | | 1.106 ± 0.07 | |  |  | | |  | |  |
| RhoA | | 0.681 ± 0.02 | |  |  | | |  | |  |
| RAC1 | | 0.807 ± 0.05 | |  |  | | |  | |  |
| SHC1 | | 1.065 ± 0.20 | |  |  | | |  | |  |
| SOS1 | | 1.249 ± 0.06 | |  |  | | |  | |  |
| SRC | | 1.333 ± 0.09 | |  |  | | |  | |  |
| TRAF3 | | 0.785 ± 0.05 | |  |  | | |  | |  |
| VEGFA | | 2.241 ± 0.09 | |  |  | | |  | |  |
